# Recurrent pyogenic cholangitis

**DOI:** 10.1002/ccr3.7584

**Published:** 2023-06-15

**Authors:** Narendra Pandit, Durga Neupane

**Affiliations:** ^1^ Department of Surgical Gastroenterology Birat Medical College Teaching Hospital (BMCTH) Biratnagar Nepal; ^2^ Department of Surgery B.P. Koirala Institute of Health Sciences Dharan Nepal

**Keywords:** cholangitis, hepatolithiasis, recurrent, stone clearance

## Abstract

The incidence of hepatolithiasis is variable. Globalization has additionally altered disease dynamics globally. A multi‐disciplinary team approach is deemed necessary for the timely diagnosis, safe, affordable treatment, and good prognosis.

A 59‐year‐old South Asian female presented with the complain of pain upper abdomen and recurrent fever for 1 year. There was no history of vomiting, jaundice, anorexia, or weight loss. Family history was unsupportive of diagnosis. On laboratory examination, alkaline phosphatase was elevated by two times. Serum bilirubin was normal, and her leukocytes count was 11,200/mm^3^. Rest of the systemic examination was normal. Contrast enhanced computed tomography (CECT) revealed multiple hypodense/hyperdense lesion in segment 3 of liver. Magnetic resonance imaging (MRI) showed hypo‐hyperdense focal lesions with conglomerate appearance on segment 3 of liver suggestive of hepatolithiasis (Figure 1). Left lateral segmental resection (Figure 2) and intrahepatic choledochoscopy from dilated left hepatic duct and stone clearances were done. On histopathological examination, definitive diagnosis of recurrent pyogenic cholangitis was established with no evidence of malignancy. At a 3‐year follow‐up, the patient is free of recurrence.

**FIGURE 1:**
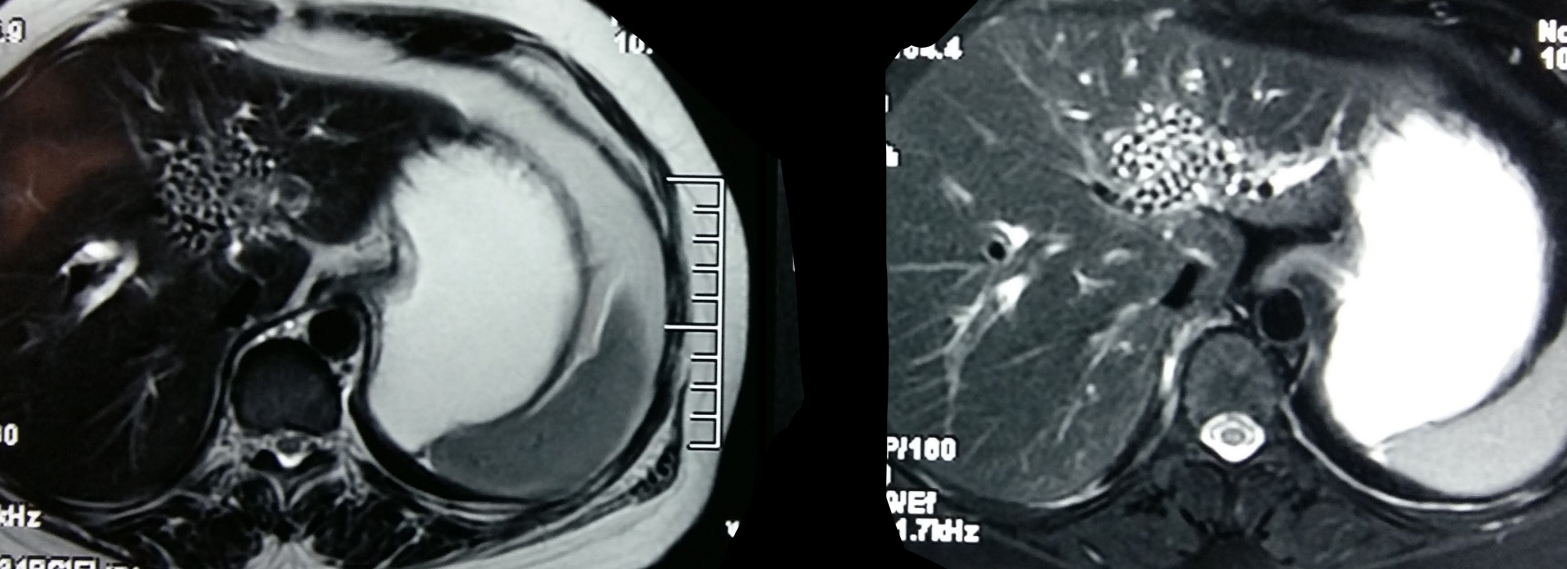
Magnetic resonance imaging (MRI)‐T2 sequence showing hypointense foci of variable sized within the left hepatic duct forming “conglomerate appearance”.

**FIGURE 2:**
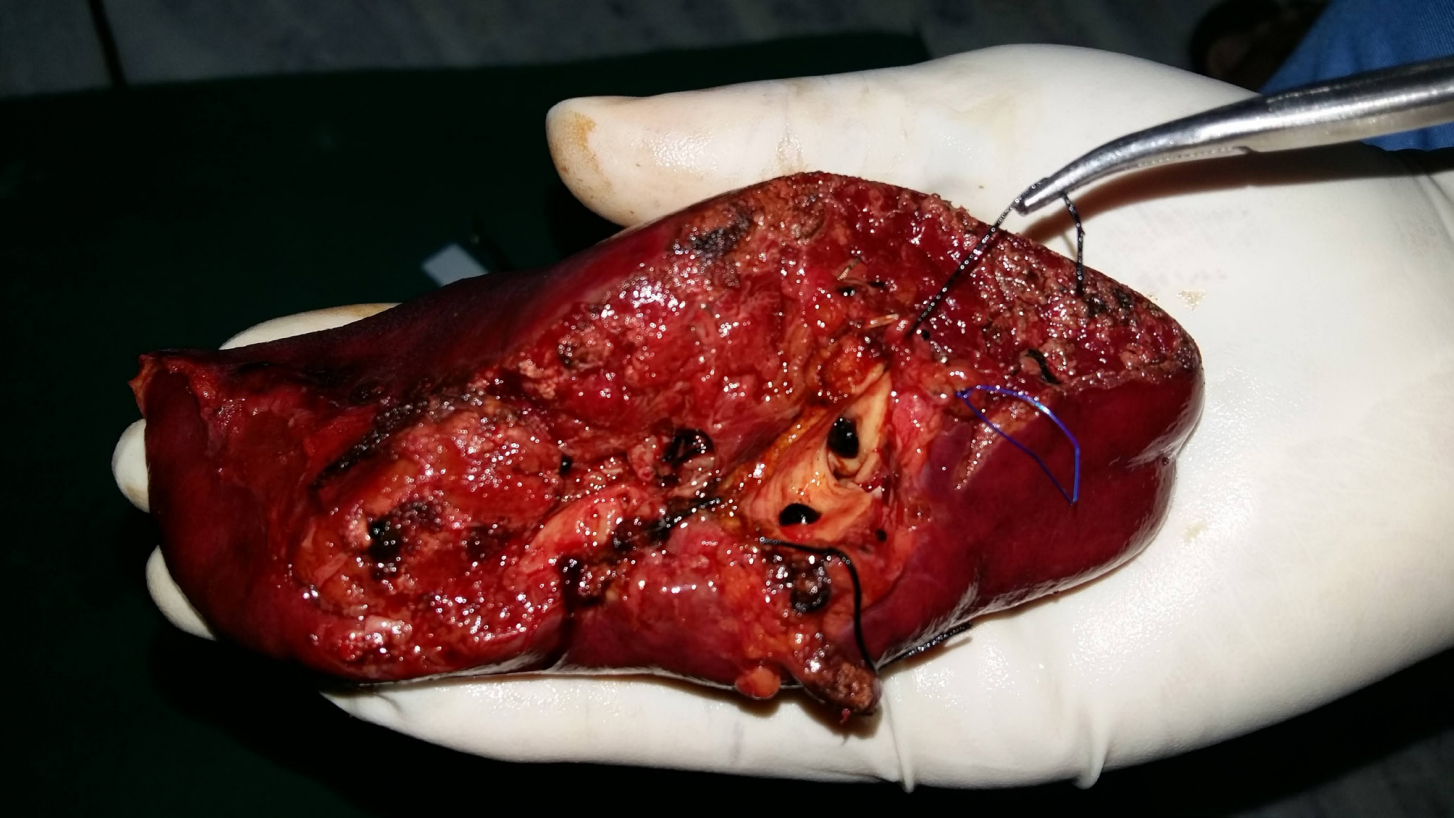
Post‐operative specimen after left lateral segmental resection of liver with multiple blackish pigmented calculi in the left hepatic duct.

The incidence of hepatolithiasis is variable. It is highly prevalent in parts of Asia, such as China, Japan, and South Korea, with a reported incidence between 3.1% and 21.2%.^1^ However, with an incidence of about 1%, hepatolithiasis is rare in Western countries. The mechanism of development of hepatolithiasis is yet to be fully elucidated. Cholestasis, cholangitis, an anatomical abnormality of the bile ducts, abnormal bile metabolism, malnutrition, and low socio‐economic status are significant risk factors for hepatolithiasis. According to several studies, indications of hepatectomy for hepatolithiasis include the following instances: (i) unilobar hepatolithiasis, and particularly that on the left; (ii) atrophy or severe fibrosis of the affected liver segments or lobe; (iii) presence of a liver abscess; (iv) cholangiocarcinoma; and (v) multiple intrahepatic stones causing marked biliary stricture or dilation.^2^ Globalization, apart from changing the socio‐economic status of regions, has additionally altered disease dynamics globally. Hepatolithiasis, as a result of recurrent pyogenic cholangitis, although still rare, is becoming progressively evident in the West because of immigration from the Asia‐Pacific region, where the disease is endemic. Such rare but emerging disease has imposed significant challenges to the physicians and surgeons. Uenishi et al.^3^ presented outcomes for 86 patients who underwent a hepatectomy from 1998 to 2012. Seventy‐six patients (88%) had immediate stone clearance whereas 82 patients (95%) had final stone clearance.^3^ A multi‐disciplinary team approach involving radiologists, internists, pathologists, oncologists, and surgeons is deemed necessary for the timely diagnosis and safe, affordable treatment, thus ensuring good prognosis to the patients.

Low socio‐economic status of our patient could have been the attributing factor. Also, unilobar hepatolithiasis and particularly that on the left was the main indication for left lateral segmental hepatectomy in our case. With this image, we would like to recommend that the differential diagnosis of hepatolithiasis should be thought in the back of mind if an elderly person from endemic region presents with the complain of pain upper abdomen and recurrent fever. Surgical intervention is deemed necessary with the aforementioned indications, and it can provide good prognosis to the patient with an excellent stone clearances.

## AUTHOR CONTRIBUTIONS

**Narendra Pandit:** Conceptualization; data curation; investigation; methodology; resources; supervision; writing – original draft; writing – review and editing. **Durga Neupane:** Conceptualization; data curation; investigation; methodology; resources; supervision; writing – original draft; writing – review and editing.

## FUNDING INFORMATION

None.

## CONFLICT OF INTEREST STATEMENT

None.

## CONSENT

Written informed consent was obtained from the patient for the accompanying images.

## Data Availability

Relevant data are available on the manuscript.
